# Creation of a pharmacogenomics patient portal complementary to an existing institutional provider-facing clinical decision support system

**DOI:** 10.1093/jamiaopen/ooab067

**Published:** 2021-08-27

**Authors:** Elizabeth Lipschultz, Keith Danahey, Tien M Truong, Emily Schierer, Samuel L Volchenboum, Mark J Ratain, Peter H O’Donnell

**Affiliations:** 1 Center for Research Informatics, University of Chicago, Chicago, Illinois, USA; 2 Center for Personalized Therapeutics, University of Chicago, Chicago, Illinois, USA; 3 Committee on Clinical Pharmacology and Pharmacogenomics, University of Chicago, Chicago, Illinois, USA; 4 Department of Medicine, University of Chicago, Chicago, Illinois, USA; 5 Department of Pediatrics, University of Chicago, Chicago, Illinois, USA

**Keywords:** patient portals, education of patients, internet-based intervention, pharmacogenetics, precision medicine

## Abstract

**Background:**

Applied pharmacogenomics presents opportunities for improving patient care through precision medicine, particularly when paired with appropriate clinical decision support (CDS). However, a lack of patient resources for understanding pharmacogenomic test results may hinder shared decision-making and patient confidence in treatment. We sought to create a patient pharmacogenomics education and results delivery platform complementary to a CDS system to facilitate further research on the relevance of patient education to pharmacogenomics.

**Methods:**

We conceptualized a model that extended the data access layer of an existing institutional CDS tool to allow for the pairing of decision supports offered to providers with patient-oriented summaries at the same level of phenotypic specificity. We built a two-part system consisting of a secure portal for patient use and an administrative dashboard for patient summary creation. The system was built in an ASP.NET and AngularJS architecture, and all data was housed in a HIPAA-compliant data center, with PHI secure in transit and at rest.

**Results:**

The YourPGx Patient Portal was deployed on the institutional network in June 2019. Fifty-eight unique patient portal summaries have been written so far, which can provide over 4500 results modules to the pilot population of 544 patients. Patient behavior on the portal is being logged for further research.

**Conclusions:**

To our knowledge, this is the first automated system designed and deployed to provide detailed, personalized patient pharmacogenomics education complementary to a clinical decision support system. Future work will expand upon this system to allow for telemedicine and patient notification of new or updated results.

## INTRODUCTION

Applied pharmacogenomics has opened up a variety of potential opportunities for improving the quality of patient care, notably via two fronts: personalized medicine (the right drug, for the right patient, at the right time) and increased patient engagement, satisfaction with care, and self-advocacy.^1^^,^^2^ However, the latter path is highly dependent on the availability, usability, and relevance of patient-targeted education resources. Pharmacogenomics presents challenges for patient education and involvement. Knowledge of genetic concepts, terminology, and data are highly variable, even among skilled professionals.^3^ For patients, who are unlikely to have any training in pharmacology or genomics, the issue is compounded.

The landscape of personalized medicine has been marked by the development of a number of online resources, electronic medical record (EMR) plug-ins, and other clinical decision supports (CDS) designed to augment prescribing practices with genetic information.^4–8^ Most educational resources in pharmacogenomics have been centered on informing care providers or counselors, with the expectation that they will be able to pass this knowledge on to their patients or clients with little or no assistance. However, attention has recently turned to possible patient education efforts such as resources for providers educating patients,^3^ patient education videos,^9^ or access to post-test genetic counseling.^10^ There are currently opportunities to further involve patients more deeply in the process of pharmacogenomics-guided prescribing, particularly in a way that is both accessible to a lay reader and fully personalized. In more general use cases, patients show extremely high satisfaction with shared EMRs,^11^ which makes the “shared record” model a promising avenue for research.

To address this lack of comprehensive patient education, we implemented the YourPGx Patient Portal to complement the existing Genomic Prescribing System CDS. The Genomic Prescribing System is a provider-facing web application that provides annotated pharmacogenomic test results to over 400 providers at the University of Chicago. The Genomic Prescribing System features CDS summaries written from a custom pharmacogenomic database, integrated with patient-specific information, and displayed on a secure website.^12^ Patients showed a significant increase in knowledge of pharmacogenomics after exploring a mockup of the YourPGx Patient Portal.^13^ The mockup was modeled closely after the Genomic Prescribing System but differed via the inclusion of additional educational information about pharmacogenomics, as well as test results written for lay readers. Promising initial results led to the further development of the application with the goal of creating a dynamically updated pharmacogenomics patient education tool and results delivery system that works in tandem with the Genomic Prescribing System. The YourPGx Patient Portal was designed to be a companion to the Genomic Prescribing System, allowing patients to view the same pharmacogenomics results as their providers, presented in an analogous format while taking into account the adjustments that must be made to support patient comprehension. Here we describe the technical details of a system that meets three criteria: (1) a web experience for patients that includes rich text formatting, intuitive report navigation, and educational imagery pertinent to their results; (2) an intuitive front-end based Create, Read, Update, and Delete (CRUD) service available to content area experts for pharmacogenomics result summaries, which are matched one-to-one with provider CDS summaries at the database level; and (3) Health Insurance Portability and Accountability Act (HIPAA) secure patient access to available results.

## METHODS

### Conceptual model

**Figure 1.**
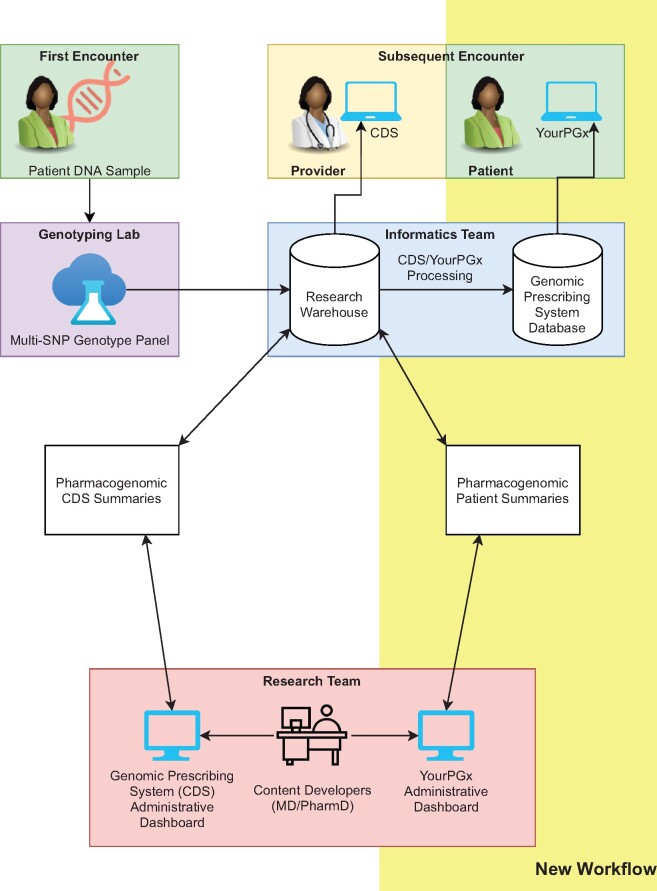
A conceptual flow diagram illustrating workflow of the parallelized provider-facing Genomic Prescribing System and patient-facing YourPGx systems. A single DNA sample for each patient is assayed and assigned genotype information, stored in a research database. Members of the research team have previously written (and currently maintain) provider-targeted CDSs that align with the available genotypes. In the new YourPGx workflow (highlighted in yellow) summaries are fed from an administrative submission form into an instance of the YourPGx summary data model, which is connected to the relevant instance of the GPS data model via a primary-foreign key relationship. That data model instance is then accessed by the YourPGx patient portal when a patient logs in to see their results.

A conceptual model of the parallelization of the Genomic Prescribing System and YourPGx is featured in Figure 1. A key design concern for the creation of YourPGx was the need of the CDS development team (consisting of MDs and PharmDs specializing in pharmacogenomics) to create patient-oriented summaries that paired one-to-one with existing Genomic Prescribing System provider summaries. This required the Genomic Prescribing System summaries to be readable from the patient summary editing interface. Also important was the creation of a new web application accessible to patients via a password-protected interface, which used an analogous application service layer to the Genomic Prescribing System in order to pair individual patients with the appropriate result summaries.

### User interface design

#### Design process

In order to design a system appropriate for patients, we invited patients who had previously agreed to participate in personalized medicine research at our institution to participate in a focus group session, to conduct initial user testing, usability assessment, and to seek specific patient stakeholder feedback on an early mockup of the YourPGx system. Patients (*n* = 20; 10 in each of two separate focus groups) were an average of 61 years of age, 60% female, 35% White/50% Black/15% Other, and had reported education levels of 20% high school or less, 25% some college, 15% college graduates, and 40% advanced degrees.^13^ Feedback collected from these patients was then directly incorporated into the design of the software, especially with respect to desired features of the user interface. We specifically focused on intuitive design tactics intended to be straightforward even for users less practiced at navigating software applications (eg, inclusive across age ranges) and those with diverse educational backgrounds: large font sizes, high contrast text, left-to-right, and top-to-bottom navigation, use of images alongside the text, and a writing style catered to readability standards. The system was further refined during an iterative process which involved regular review of software interface design by physician scientists, web developers, and other medical professionals.

#### Patient application user interface

The goal of the YourPGx Patient Portal user interface is to safely educate patients about pharmacogenomics, allowing them to interface with personalized pharmacogenomics results in a way that will facilitate conversation about pharmacogenomics with providers. To address this goal, key design priorities for the patient portal included accessibility for individuals with varying degrees of internet literacy, HIPAA-compliant security in-transit and at rest, and a clear visual hierarchy for increased navigability and comprehension.

We designed a website using the bootstrap front-end framework to guide patients through initial educational materials via a visually prominent top of the page menu bar. Using a single-page application, educational summaries for any drugs for which a patient had actionable pharmacogenomic test results were presented to patients, regardless of whether the patient had been prescribed those drugs. The summaries were presented in both a compact format suitable for printing, and an expanded page presented in a list format with detailed descriptions embedded in an accordion interface. These descriptions intended to support bulleted summaries of established clinical findings in pharmacogenomics related to their genotype and recommended talking points for discussion with their providers. They also are able to support embedded images in order to allow for graphically augmented explanations of complicated topics such as pharmacogenomics effects on metabolic processes and variant prevalence in the population.

The patient user interface was created as a standalone web application to be accessible to patients from within the institutional network, consistent with other existing patient portals.^14^ For the first iteration of actual patient use of the finished portal, we chose a cohort of hospitalized inpatients who had already enrolled to participate in one of our institutional, IRB-approved pharmacogenomic testing projects (clinicaltrials.gov #NCT03225820). For this initial clinical offering, login to the page was limited to the study team and authenticated via university network credentials. Team members were then able to select the patient who would be viewing their results. This allowed the study team to conduct “guided” one-on-one sessions with patients as they explored the portal, so as to further monitor initial user adoption, comprehension, and feedback. Future iterations of the portal will allow for individual sign-on by patients via a new TLS protocol.

#### Administrative content creator user interface

The administrative user interface was designed as part of the existing administrative dashboard, which is built in the ASP.NET Model-View-Controller framework using RESTful API calls from AngularJS controllers in order to facilitate quickly loading and dynamically updating content. Design priorities included straightforward tools for the creation, previewing, and updating of patient-oriented summaries as well as the ability to compare patient summaries to the analogous Genomic Prescribing System decision support. Also important was the ability to create patient summaries with a variety of information layouts, including uploaded images. Additionally, the ability to draft summaries, mark them for review, and “push” them to a live status was necessary. Ajax calls to the backend allowed for the automatic saving of summaries during the writing process, as well as frequent updates in case two users happened to have the same summary open in the editing deck simultaneously. All text editing interfaces were built with ckeditor^15^ to allow for rich text in patient summaries. User roles tied to authenticated identities allow an editorial supervisor final control over the release of new summaries, or re-release, following updates.

### Database design

The existing Genomic Prescribing System database architecture facilitates a variety of different types of pharmacogenomic CDSs (summaries based on one or more single-nucleotide polymorphisms [SNPs], or on a more complex multiallelic phenotype), which are each housed in tables designed to relate summary information to the associated genotypes and phenotypes. When a patient’s DNA is analyzed, the genotypic information is imported into the database and mapped to the appropriate CDS summaries.

**Figure 2.**
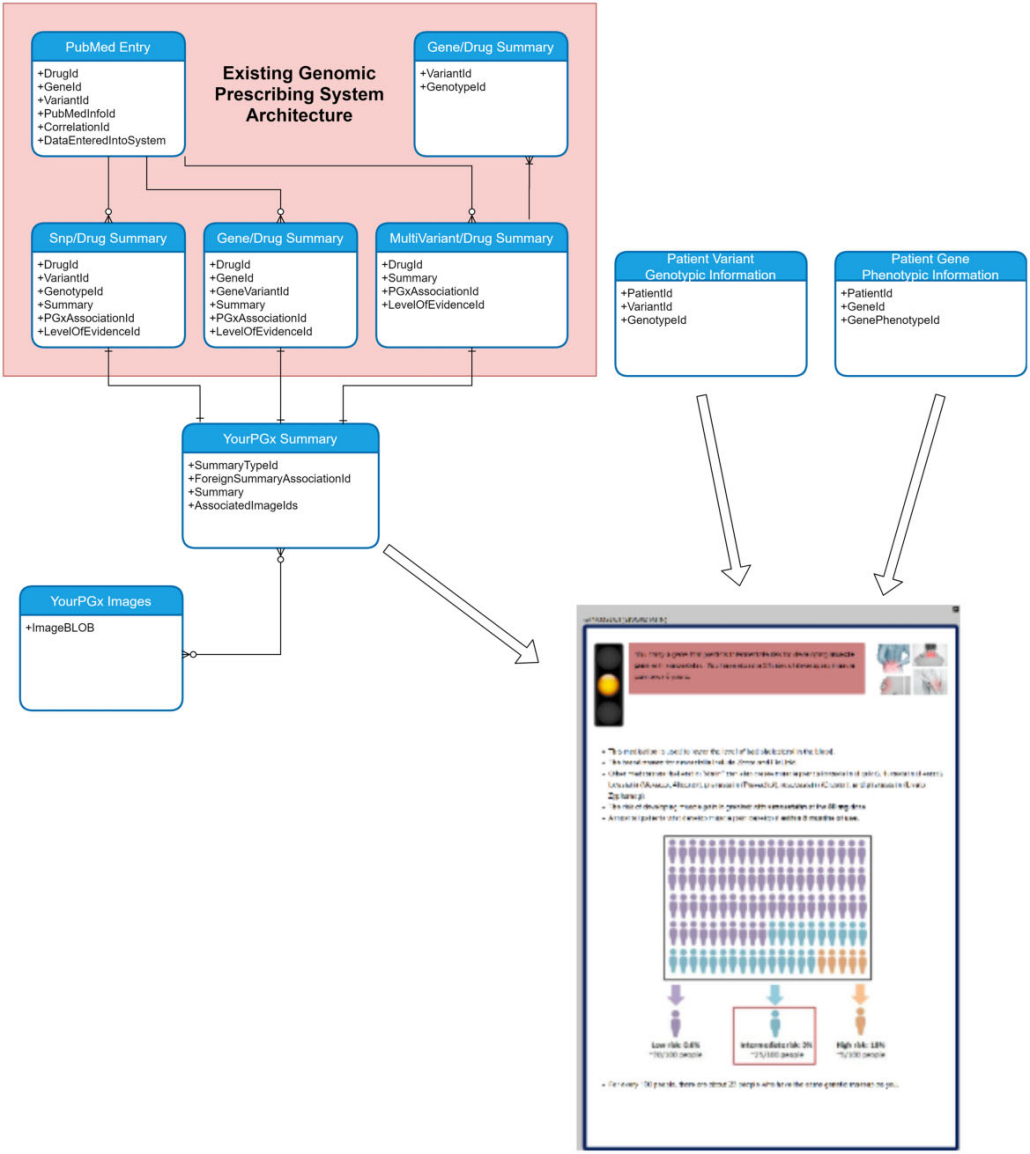
The relationship between the Genomic Prescribing System and YourPGx summaries. The Genomic Prescribing System Summaries are separated into three classes (single SNP, gene phenotype, and multivariant) which require differing table structures. As a result, two foreign keys are required to pair a YourPGx summary with a Genomic Prescribing System summary. YourPGx Summaries also include supplemental embedded images.

To facilitate the YourPGx conceptual model, we needed to relate the newly created patient summaries to existing Genomic Prescribing System summaries via a primary-foreign key relationship (Figure 2). This allowed the service that delivers summaries to patients on the YourPGx front-end to use the same core logic as the service that delivers summaries for the Genomic Prescribing System. It also supported the key Genomic Prescribing System-YourPGx one-to-one relationship. Techniques of database normalization were used where appropriate, such as 3NF schematized storage of the BLOB-encoded images used in patient summaries to avoid large-scale data duplication.

### Security

Login to the administrative dashboard is managed by University of Chicago web services, and access is controlled by the University of Chicago Biological Sciences Division (BSD) Information Security Office.^16^ Both a BSD account and Center for Personalized Therapeutics (CPT) Informatics team approval are required for login. All data are stored in a database housed at our HIPAA compliant Kenwood Datacenter, managed by the University of Chicago Center for Research Informatics (CRI).^17^ Login to the patient portal leverages the same system. It is only possible to log into either service via the institutional network locally or via VPN.

### Logging

Services in both the administrative dashboard and the patient portal can log user actions, tied to a user ID and timestamp, allowing for usage analysis of both applications. Login to the portal and navigation throughout the portal can be analyzed to provide insight into which subpages are most frequently visited, how long is spent on subpages or from login to logout, and what results are viewed. This was accomplished via a custom logging system built for research purposes, rather than an out-of-the-box error logging system, to allow for greater flexibility.

## RESULTS

### Deployment

#### Results formatting

**Figure 3:**
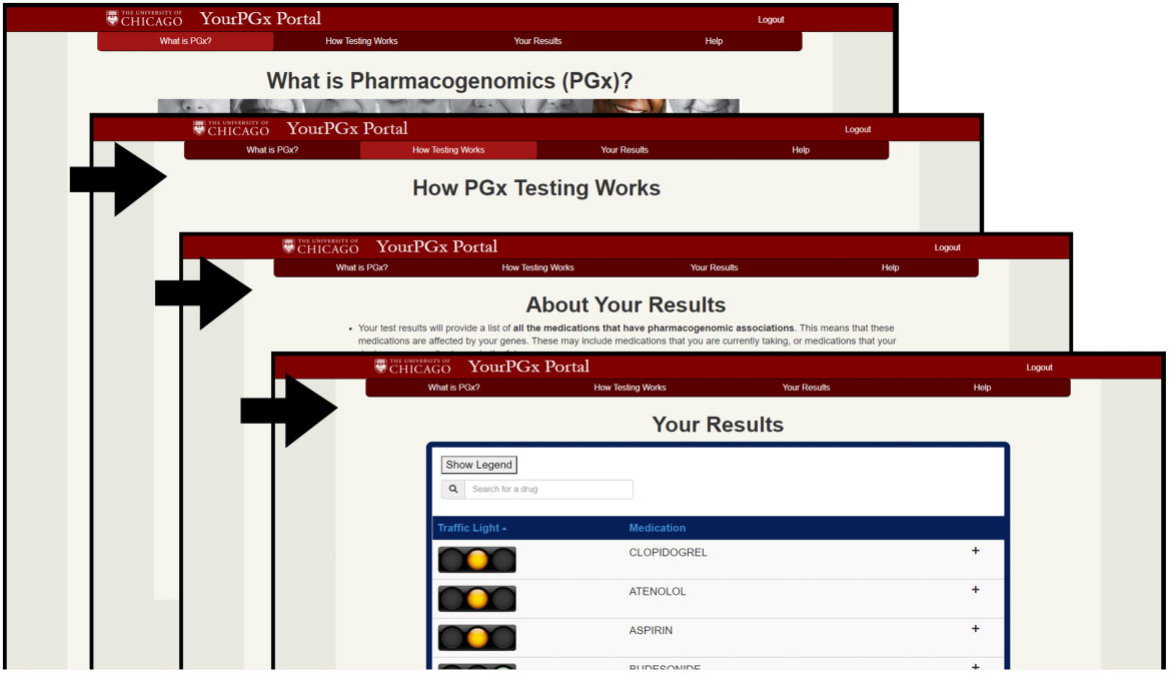
Top of page tabs allow for navigation to educational resources which contextualize results for patients, with a left-to-right format guiding users through a prescribed course before leading them to a results page. A legend which defaults to open but can be hidden serves as a key for the traffic light iconography as it pertains to Pharmacogenomics. Results are available in a compact format, but are expandable to reveal detailed information about the medication written specifically for this phenotype by content developers.

Hierarchical webpage design featuring a simple menu bar at the top of the page allows for natural left-to-right navigation through the content of the website (Figure 3). The single-page application (SPA) design of the results page allows for quick navigation between summaries via the JavaScript-enabled dynamic results panels that expand and retract to reveal and hide summary details without refreshing the page. As more summaries become available for a patient over time, they are automatically integrated into this panel.

#### Access

The YourPGx Patient Portal is currently available to patients in the inpatient setting who have been previously genotyped for the Genomic Prescribing System via hospital-provided iPad. Login is facilitated by a research coordinator as part of a clinical study of patient behavior in the portal. All clicks from the patient login are recorded in the research database to facilitate future insight into patient use of the application.

### Content developer user interface

The conceptual model allowed for the creation of a system that supports one-to-one matching of YourPGx summaries with Genomic Prescribing System summaries. Creating a structure such that fully independent YourPGx summaries relate one-to-one with Genomic Prescribing System summaries allows the overall format of results to be preserved while providing greater flexibility for patient content developers to configure summaries which are appropriate for patient education.

**Figure 4.**
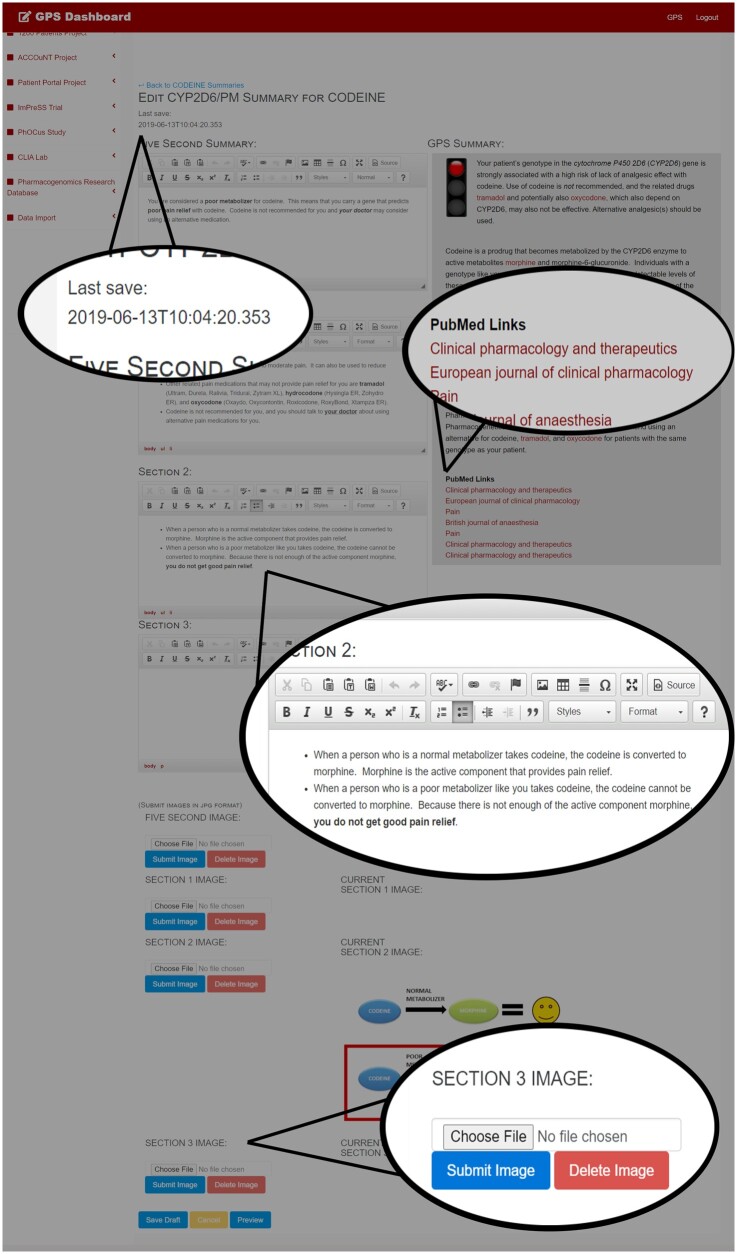
The content developer editing interface. All available Genomic Prescribing System summaries are available to be paired with a companion YourPGx summary. The editing interface is equipped with multiple rich text editing sections for content writing and multiple upload locations for the inclusion of embedded images. The editor automatically saves drafts of the patient summary for future work, and provides a side-by-side view of the editing interface and the existing Genomic Prescribing System summary and links to referenced sources to allow content developers to reference the existing information available to providers for this phenotype while creating the patient summary.

The YourPGx content developer area of the administrative dashboard allows CRUD functionality for the drafting and release of fully featured YourPGx summaries that complement the existing Genomic Prescribing System summaries (Figure 4). The interface features multiple rich-text entry boxes and image upload buttons that allow for modular summaries with up to four images interspersed amongst four sections of text. The content autosaves periodically while the developer is active in the interface, preventing lost work. The editing interface is accompanied by a read-only view of the existing Genomic Prescribing System summary that the YourPGx summary is being written to accompany. This view includes live links to the relevant literature that was cited in the Genomic Prescribing System summary, to allow the content editor to leverage the existing knowledge base while writing. User roles within the administrative dashboard allow members of the content development team varying privileges, depending on individual assigned roles. Users were designated either as a content writer (with privileges to create or edit summary drafts) or content reviewer (with privileges to edit, approve, or reject summary drafts), and these roles were stored in the research database and checked at each login.

At the time of writing, there are 58 unique summaries corresponding to drug-genotype/phenotype pairings, which consist of information across 10 drug classes (Table 1). With the content developer user interface, this number will be expandable to encompass patient complements to the full range of summaries available in Genomic Prescribing System.

**Table 1.** Current coverage of the YourPGx Patient Portal

Drug class	Unique summaries	Available to *n* = 544 patients
Steroid	9	456
Nonsteroidal anti-inflammatory	3	447
Narcotic analgesic	4	399
Beta-blocker	9	459
Antiplatelet	4	403
Proton pump inhibitor	15	1612
Statin	6	916
Vasodilator	7	Not yet assigned
Serotonin 5HT3 receptor antagonist	1	Not yet assigned
Total	58	4692

### Initial application to clinical population

As of July 2020, the applicability of this tool is being explored in an existing IRB-approved clinical study of 544 previously hospitalized patients genotyped as part of the African American Cardiovascular Pharmacogenomics Consortium (ACCOuNT).^18^ Physicians, midlevel providers, and pharmacists associated with those patients have access to personalized pharmacogenomic prescribing guidelines through the Genomic Prescribing System. We matched available clinical genotype results to the existing YourPGx summaries and validated this process for 100% of matches programmatically, as well as manually in a 5% sample group of patients, by checking that each YourPGx summary available to an individual patient was consistent with the results available to that patient’s providers in the Genomic Prescribing System. This was done to ensure consistent delivery of accurate results. This application of YourPGx demonstrated 4692 potential summaries that could be available to this patient set. Security of the system was independently verified by a subset of researchers working on the project, and completeness of logging has been demonstrated in the initial YourPGx test cohort.

## DISCUSSION

This study demonstrated the feasibility of extending the phenotypic assignment logic of a preexisting CDS system to support a patient-oriented educational web platform. We also explored the role of a robust content developer user interface in marrying a CDS system with a patient portal. Building on the framework of that union, we created and deployed a first-of-its-kind interactive pharmacogenomics patient results web portal. This work sets a precedent for future interactive, personalized patient pharmacogenomics systems, and we hope it can serve as a model for other institutions. This work also lays the ground for the future development of CDS-adjacent patient education systems.

The possibility to create a pharmacogenomics patient education and results delivery system that works as a technical extension of a CDS system invites new opportunities for more comprehensive patient education. Other research has highlighted the importance of patient education in the pharmacogenomics implementation process,^19^^,^^20^ and some have begun to advance solutions for patient education within this realm.^21^ In addition, other research has discussed simultaneous provider and patient education as a possible framework to produce improved clinical outcomes.^22^ The recent expansion of entities providing genetic education and results reporting to patients^23–27^ is likely to only accelerate needs for patient genomic education tools. However, the YourPGx portal sought a deeper level of provider–patient harmonization to an educational process through its novel CDS extension-based development, allowing providers and patients to simultaneously receive pharmacogenomics educational material personalized to the patient, and presented at appropriate levels of detail for both a patient and a provider, through complementary web applications. Within this framework, providers receive CDS that provide in-depth quantitative data about published patient outcomes regarding the pharmacogenetic association, specific prescribing recommendations, and strength-of-evidence scores. Patients instead receive summaries which focus on likelihood of side effects or medication inefficacy (for them, compared to the general population), basic explanations of how pharmacogenomic factors may impact medication metabolism, and questions to ask their providers.

This study also showed the feasibility of readily extending a few unique patient educational modules to thousands of actionable genomic results in a population of hundreds of patients. One-to-one matching of provider CDS summaries with patient pharmacogenomics summaries further expedites the creation of patient results by allowing content developers to model patient education for a gene–drug combination on provider education for that same phenotype. Further, a robust content development suite that includes tools for viewing existing provider CDS summaries, referencing relevant publications, formatting the layout of patient summaries, and uploading educational imagery can help to ease the process of creating these results.

Our initial implementation of YourPGx focused on hospitalized inpatients, but this was chosen only by convenience in that such patients provided a more natural audience with which to test and garner feedback on the use of the system without the more intense time constraints imposed by the outpatient or perisurgical settings. Indeed, our initial implementation utilized one-on-one “guided” accessions of a patient’s YourPGx portal with the bedside assistance of a clinical research staff member who helped the patient navigate the system (each session typically lasted about 20 minutes). It is acknowledged that such involvement of the research team member in the assisted navigation of the portal for the initial pilot deployment may have introduced bias in the usability assessments from this specific setting; however, this pilot deployment program occurred after the design process had been completed. In contrast, the initial patient-user testing which occurred during design development (as part of the focus groups) was unassisted and unguided. Future implementations additionally are already planned to expand to the outpatient and presurgery settings, whereby patients will be given access to their YourPGx portal through a unique electronic prompt or “invitation”—without the real-time guidance of a staff member. This is similar to patient self-accessions of their own medical records/results (eg, EPIC “MyChart” feature), which are now commonplace and increasingly viewed as standard offerings. The increasing patient comfort and familiarity with using such contemporaneous self-access portals likely will only strengthen the future adoption of complementary applications like the YourPGx portal.

This application has limitations. For the scope of this initial release, the portal is solely available within the University of Chicago Medical Center network and is not currently accessible to patients through remote access. Our model of deployment presently functions via provider-guided navigation on institutionally provided iPads, a choice that has allowed for observation of patient experience during the initial phase of clinical implementation. However, options are being explored for extending access to patients remotely in a future version, which is an important adaptation to the telemedicine-mediated ecosystem. The system also does not yet support patient notification of available results (eg, via “MyChart”), but this feature can be crafted to correspond to existing best practice alerts in the Genomic Prescribing System intended to notify users of new information. The YourPGx Portal currently does not have an application available for download on iOS or Android marketplaces, but the website has been optimized for mobile browser navigation. The future creation of a mobile application may be an appealing solution, especially for patients more adept with mobile technologies. Additionally, the system was built as an extension of the extant institutional genomic prescribing system. Replication of this project could involve the ground-up construction of a gene-to-phenotype matching system but could also be facilitated via the creation of an API to access another database of pharmacogenomics test results. One option for this could be to collect data from direct-to-consumer pharmacogenetic test results.

## CONCLUSIONS

We have created a novel web application and development suite that extends the relational database and API of an extant CDS system to create a patient-oriented education and results delivery system. The system was created with features that improve the accessibility of pharmacogenomics information to patients and families via descriptive imagery and lay-oriented complementary summaries of CDS results. These patient summaries can be created and modified in-production via a set of dedicated content developer tools integrated into an administrative dashboard. Future work will expand upon this system to allow for telemedicine and patient notifications, as well as increasing the content base to cover additional medications and their associated actionable variants to further support clinical pharmacogenomics decision-making processes.

## CONTRIBUTORS

E.L. was the primary software developer of the patient portal and administrative dashboard, analyzed the data, and drafted the manuscript.

K.D. provided development support, and was the primary developer of the pre-existing decision support system with which the portal and dashboard integrate. KD also provided data and helped draft the manuscript.

T.M.T. advised on user interface design, developed content for the portal, analyzed data, and edited the manuscript.

E.S. provided user interface design support, performed initial manual validation of system mappings, and edited the manuscript.

S.L.V. provided resources for development and maintenance of the portal, and edited the manuscript. M.J.R. provided resources for development and maintenance of the portal, and edited the manuscript. P.H.O. was conceptual originator of the idea, provided resources for development and maintenance of the portal, advised user interface design, developed content for the portal, analyzed data, and helped draft the manuscript.
